# Socio-demographic predictors of the prevalence of dysfunctional breathing in a healthy population during the COVID-19 pandemic

**DOI:** 10.1192/j.eurpsy.2022.1256

**Published:** 2022-09-01

**Authors:** J. Koniukhovskaia, E. Pervichko, V. Petrenko, O. Mitina, O. Stepanova, I. Shishkova, E. Dorokhov

**Affiliations:** 1 Pirogov Russian National Research Medical University, Clinical Psychology Department, Moscow, Russian Federation; 2 Lomonosov Moscow State University, Psychology, Moscow, Russian Federation; 3 Ryazan State Medical University named after I.P. Pavlov, Faculty Of Clinical Psychology, Ryazan, Russian Federation

**Keywords:** Covid-19 pandemic, dysfunctional breathing

## Abstract

**Introduction:**

Dysfunctional breathing is a breathing patterns that do not correspond to the physiological needs of the body, provoke many poly-systemic symptoms. Dysfunctional breathing is experienced as a feeling of “difficulty in breathing”, which in the conditions of the COVID-19 pandemic may be similar to the symptoms of coronavirus infection (Taverne et al., 2021).

**Objectives:**

To examine the role of socio-demographic predictors in the prevalence of dysfunctional breathing in the Russian population during the COVID-19 pandemic.

**Methods:**

The author’s socio-demographic questionnaire, the Naimigen Questionnaire (Van Dixhoorn, Duivenvoordent, 1985), the STAI (Spielberger et al., 1983) and the “Perceived Stress Scale-10” (Cohen,Kamarck,Mermelstein,1983) were used. The study was conducted online from April 27 to December 28, 2020. It was attended by 1,362 people from all regions of Russia (38.3 ±11.4 y.o.).

**Results:**

In men, the average values for NQ (11.19±7.74) are lower than among women (18.73±9.96, p=0.000). Persons with incomplete higher education have a higher score on NQ (N=103,NQ=20.44±11.8) than persons with higher education (N=1051,NQ= 17.40±9.63,p=0.048) and candidates/doctors of sciences (N=97,NQ= 15.34±11.20,p=0.005). There was also a connection between the severity of dysfunctional breathing and the level of income, which is associated with a negative correlation between income level and perception of stress (r=-0.215,p=0.000), state (r=-0.165,p=0.000) and trait anxiety (r=-0.127,p=0.000).

**Conclusions:**

The severity of dysfunctional breathing is associated with gender, income levels and education, what can be used to identify a group of people who are most susceptible to the occurrence of dysfunctional breathing during the pandemic COVID-19. The study was supported of the Russian Science Foundation, project No.21-18-00624.

**Disclosure:**

The study was supported of the Russian Science Foundation, project No. 21-18-00624.

